# Draft Genome Sequences of Strains TRE 1, TRE D, TRE H, and TRI 7, Isolated from Tamarins and Belonging to Four Putative Novel *Bifidobacterium* Species

**DOI:** 10.1128/genomeA.01449-17

**Published:** 2018-01-18

**Authors:** Edoardo Puglisi, Paola Mattarelli, Monica Modesto, Andrea Bonetti, Caterina Spiezio, Camillo Sandri, Lorenzo Morelli

**Affiliations:** a Department for Sustainable Food Processes, Università Cattolica del Sacro Cuore, Piacenza, Italy; b Department of Agricultural Sciences, Università di Bologna, Bologna, Italy; c Parco Natura Viva, Garda Zoological Park, Bussolengo, Italy

## Abstract

*Bifidobacterium* sp. strains TRE 1, TRE D, TRE H, and TRI 7 were isolated from two tamarins housed in Parco Natura Viva, Garda Zoological Park S.r.l. (Bussolengo, Verona, Italy). These strains belong to four putative novel species of the genus *Bifidobacterium*. The genome sizes were 2.7 Mb for TRE 1, 2.7 Mb for TRE D, 2.4 Mb for TRE H, and 2.7 Mb for TRI 7. The average GC contents were 63.18% for TRE 1, 58.27% for TRE D, 57.11% for TRE H, and 63.79% for TRI 7.

## GENOME ANNOUNCEMENT

Bifidobacteria are Gram-positive bacteria with high G+C contents that are commonly found in the human and animal gastrointestinal tracts. Bifidobacteria are considered key commensals in host-microbe interactions, and they are believed to play a crucial role in nutrition, immunomodulation, and resistance to infection. Studies on bifidobacteria from nonhuman primates have grown in recent years. Novel species within the genus *Bifidobacterium* isolated from the cotton-top tamarin (*Saguinus oedipus*) (1) and from the emperor tamarin (*Saguinus imperator*) (2, 3) have recently been reported.

Here, we report the draft genome sequences of four putative novel bifidobacterial strains; three were isolated from the feces of an adult subject of the cotton-top tamarin (strains TRE 1, TRE 9, and TRE H), and one (TRI 7) was from the feces of an adult subject of the emperor tamarin. Strains were grown in MRS plus 0.05% cysteine at 37°C under anaerobic conditions using the Anaerocult system. Under *in vitro* culture conditions, these strains were considered oxygen tolerant and microaerophilic. Cells were lysed with SDS/lysozyme treatment, and genomic DNA was extracted with a NucleoSpin tissue kit (Macharey-Nagel, Duren, Germany) according to the manufacturer’s instructions. Illumina MiSeq V3 technology for 2 × 300-bp paired-end reads was used to sequence the whole genomes (Bio-Fab s.r.l., Rome, Italy). SPAdes version 3.11 (4) was used to assemble the whole-genome sequences. The draft genome sequences were annotated using the NCBI Prokaryotic Genome Annotation Pipeline (5) and the Rapid Annotation using Subsystems Technology (RAST) server (6).

Summary data for the genome assemblies are reported in Table 1. Average nucleotide identity (ANI) analysis results between the four studied strains were always below 82.07%; two strains displaying an ANI value ≤95% are considered to belong to two distinct species. 16S rRNA sequence comparisons with all publicly available *Bifidobacterium* sequences showed a level of similarity below 97% for all four strains, with the only exception being strain TRE H, which had 99% DNA sequence similarity to *Bifidobacterium callitrichos* DSM 23968^T^. However, the genetic relatedness at the genomic level of this strain calculated by ANI analysis showed a level of identity of 77.82% with its nearest phylogenetic neighbor, *B. callitrichos*.

**TABLE 1:** Summary data of the genome assembly for the four putative novel *Bifidobacterium* species

Strain	Isolate source	No. of contigs	Genome size (bp)	GC content (%)	No. of coding sequences	GenBank accession no.
TRE 1	*Saguinus oedipus*	21	2,698,692	63.18	2,236	PEBI00000000
TRE D	*Saguinus oedipus*	22	2,652,367	58.27	2,171	PGLQ00000000
TRE H	*Saguinus oedipus*	34	2,386,956	57.11	1,931	PEBJ00000000
TRI 7	*Saguinus imperator*	48	2,723,401	63.79	2,241	PEBK00000000

The similarities of the four genomes with all publicly available genomes were also calculated with Mash/MinHash genome distance estimation implemented in PATRIC version 3.5.0 (7); the distance was always above 0.11. These data thus indicate that *Bifidobacterium* sp. strains TRE 1, TRE D, TRE H, and TRI 7 belong to four putative novel species, the full descriptions of which are still in progress.

### Accession number(s).

The assembled draft genome sequences generated in this study can be found in DDBJ/ENA/GenBank under the accession numbers reported in Table 1.
